# The mediating role of anxiety and depressive symptoms on the relationship between physical limitations and cognitive impairment among older adults in China: differences based on religious perspective

**DOI:** 10.3389/fpsyg.2025.1662166

**Published:** 2026-01-13

**Authors:** Yi Zhang, Jinhua Guo, Lixia Lin, Min Peng, Jiaxi Huang, Yi Yang, Tiemei Shen

**Affiliations:** 1 Department of Cardiovascular Disease Institute, Guangdong Provincial People's Hospital (Guangdong Academy of Medical Sciences) Southern Medical University, Guangzhou, China; 2 Department of Guangdong Institute of Geriatric Medicine, Guangdong Provincial People's Hospital (Guangdong Academy of Medical Sciences) Southern Medical University, Guangzhou, China; 3 Department of Nursing, Guangdong Provincial People's Hospital (Guangdong Academy of Medical Sciences) Southern Medical University, Guangzhou, China

**Keywords:** basic activities of daily life, cognitive impairment, mediating effect, mental health, path analysis, religious belief

## Abstract

**Background:**

Physical limitationsand mental health may have a common effect on cognitive function. However, little is known about how religion influences these paths among older Chinese adults.

**Objective:**

This study investigated how anxiety and depressive symptoms mediate the association between physical limitations and cognitive impairment. Furthermore, we analyzed discrepancies in path models between participants with or without religious beliefs.

**Methods:**

This population-based, cross-sectional study involved 6,656 adults aged ≥ 60 years in six cities in Guangdong Province, China. Participants were divided into two groups based on the presence of religious beliefs. All participants completed a general demographics questionnaire, the Chinese version of the Mini-Mental State Examination (MMSE), the Basic Activities of Daily Living (BADLs) scale, Generalized Anxiety Disorder-7 (GAD-7), and Health Questionnaire-9 (PHQ-9). Simple and serial multiple mediation models were then tested using SPSS PROCESS macro.

**Results:**

BADLs, anxiety symptoms, depressive symptoms, and cognitive impairment were significantly related (all *P* < 0.01). When adjusting for sociodemographic and health-related factors, BADLs limitations had a direct positive effect on cognitive impairment among participants with religious beliefs [effect = 0.107, 95% confidence interval (CI): 0.095, 0.121], but also had indirect effect via independent suppressing of anxiety symptoms (effect = −0.005, 95%CI: −0.010, −0.002). For participants without religious belief, BADLs limitations had a direct positive effect on cognitive impairment (effect = 0.135, 95%CI: 0.129, 0.140) as well as an indirect effect via three paths: an independent suppressing effect of anxiety symptoms (effect = −0.002, 95%CI: −0.004, −0.0001), independent mediation of depressive symptoms (effect = 0.003, 95%CI: 0.002, 0.004), and serial mediation of anxiety and depressive symptoms (effect = 0.004, 95%CI: 0.003, 0.006).

**Conclusion:**

Our findings highlight the need for health professionals to promote mental well-being to prevent cognitive decline prevention among older adults with physical limitations who do not have religious belief. These findings should be further confirmed by prospective studies utilizing other methods of assessing cognitive function and religiosity.

## Introduction

As global social structure changes, numerous countries, including China, are facing an aging problem and associated health issues (Qiao et al., 2022; Han et al., 2022). Cognitive dysfunction is regarded as a normal neurological change that accompanies the physiological aging process (Jia et al., 2020; Fiocco and Yaffe, 2010). However, persistent decline in cognitive function may develop into dementia or Alzheimer’s disease, leading to impaired self-care abilities and social interactions (Jia et al., 2020; Semkovska et al., 2019; Hu et al., 2019) and ultimately imposing a great burden on the public health system and healthcare financing (Jia et al., 2020; Hu et al., 2019; Ramaprasad et al., 2015). At present, the prevalence of cognitive impairment in China is higher than that of other developed countries. In European countries, nearly 10% of older adults experience cognitive decline or dementia (Caffò et al., 2022; Lara et al., 2016). In China, various regional population-based surveys report a prevalence rate exceeding 20% (Qin et al., 2022; Ding et al., 2015; Jiang et al., 2021). Hence, there is an urgent need for comprehensive and active interventions for older adults with cognitive issues.

Prior studies have investigated the association between physical function and cognitive impairment among older adult. In their prospective survey following older Japanese adults for 4 years, Sauvaget et al. (2002) found that dementia was a strong predictor of limited basic activities of daily living (BADL) or instrumental activities of daily living (IADL) after adjusting for age, sex and history of stroke. The predictive value of changes in cognition to disability has been confirmed by several longitudinal studies. Exploration of the potential mechanism connecting cognitive impairment with physical function has become an important research topic. Wu (2021) reported that good baseline cognitive function decreased the risk of endpoint BADL disability over a 2-year follow-up period, and that depressive symptoms mediated the negative effect of cognitive function on BADLs. Using longitudinal data from the Chinese Longitudinal Healthy Longevity Surveys, Li and Wu (2022) found that 29.1% of older adults with cognitive impairment developed IADL disability in 6 years and that the correlation was fully mediated by lifestyle, social communication, and depressive status. Mental disorders are crucial mediators accelerating functional decline among older adults with limited cognitive competence (Peng et al., 2023; de Paula et al., 2015). This effect may be due to a synergistic link between cognitive impairment and depressive status, such that neuroinflammatory or morphological changes in the brain lead to impaired executive control and mood processing (Naismith et al., 2012; Butters et al., 2008). Mechanistic studies suggest that these effects might derive from bodily damage (Peng et al., 2023), indicating a possible bidirectional correlation between physical and cognitive impairment via the same influencing path (Sun et al., 2022). Moreover, Panza et al. (2010) reported that mental diseases like depression may be a reaction in initial symptoms of cognitive decline and show similar behavior features of clinical function impairment, suggesting a vital overlap or interaction effect (Zhang and Yang, 2024; de Paula et al., 2015). Based on this background, we hypothesized that mental disorders like anxiety or depression play an indirect role in the ADLs disability—cognitive function association.

Several factors are recognized as possibly protective against cognitive decline, including healthy living habits (Bauman et al., 2016) and dietary patterns (Aune et al., 2017), positive or optimistic emotions (John et al., 2019), frequent social activities (Li and Wu, 2022), and specific religious faiths or beliefs (Hosseini et al., 2019). The effect of religiosity or religious practice on delaying cognitive development—particularly dementia—has attracted great research attention in the past decade. According to a systematic review of 17 studies (Hosseini et al., 2019), a positive association between religious/spiritual practice and cognitive function was reported in 82% of studies. Nath et al. (2023) also carried out a systematic review to explore the role of religion on cognitive maintenance, demonstrating significant consistency in the direction of the relationship between religion and memory, suggesting that region is a key element of cognitive protection among middle-aged and older adults. Prior studies have suggested several mechanisms to explain the positive impact of religion on cognitive deterioration. Some viewpoints argue that religious participation can improve the formulation of nonrepresentational concepts like moral sense and meaning of life, which are beneficial for stimulating and maintaining higher cortical functions and brain regions associated with memory (Koenig, 2012). Alternatively, religion can be linked to a positive effect on health by increasing social interactions that encourage individuals to consolidate friendships and expand social networks (Bourassa et al., 2017; Kuiper et al., 2016), thus avoiding negative mental health consequences and cognitive deficits (Croezen et al., 2015). Social isolation has been shown to be a risk factor for cognitive decline in previous studies of older adults (Evans et al., 2019; Taylor et al., 2018). At the same time, the social characteristics of religious activity could strengthen cognitive reserve capacity and elevate mental tenacity (Kuiper et al., 2016; Barnes et al., 2004).

There is a broad consensus that positive religious beliefs or involvement are an important coping resource for mental health. Compared to people without religious beliefs, individuals who believe in god or have higher religiosity may have greater self-control to seek divine forgiveness, which is a potential mechanism linking religion to psychological health (Maranges and Fincham, 2024). These findings are supported by increasing data from longitudinal studies (Braam and Koenig, 2019) showing a significant association between religious belief and modest improvement of depressive symptoms over time. The necessity to combine faith-adapted mental- and cognitive-related treatments with psychological interventions for depression patients has been emphasized in multiple studies (Ceramidas, 2012; Anderson et al., 2015; Ojagbemi and Gureje, 2020). According to estimates from the Religious Blue Book China Report (2015) published by the Chinese Academy of Social Sciences, approximately 23.05 million individuals in China identify as Christian, accounting for about 1.8% of the total population, whereas Buddhists constitute roughly 18% of the population. The report further indicates that health-related concerns serve as a significant motivational factor influencing religious affiliation and participation among older adults. While cultural and philosophical foundations shape distinct patterns of spiritual belief, religious engagement, and moral orientation in Chinese society—differentiating it from Western contexts (Zhang et al., 2024; Muhammad, 2022)—the role of religion in promoting positive health outcomes has gained increasing scholarly recognition. Empirical evidence supports the notion that religious beliefs meaningfully influence daily life among adherents, contributing to improved psychological well-being and potentially mediating physiological and cognitive health trajectories (Koenig, 2015). Consequently, investigating the mechanisms through which religious beliefs associate the “physiological–psychological–cognitive” pathway in elderly populations in China may offer valuable insights for refining cognitive and mental health intervention models. Furthermore, there is a lack of research focusing on how religiosity affects the association between physical dysfunction and cognitive decline mediated by psychological factors (Zhang et al., 2024), limiting the understanding and applications of religious theories by healthcare professionals when handling cognitive issues.

This study is grounded in the theory of life meaning—a foundational theory within positive psychology that posits the search for meaning in life as a fundamental human motivation. First articulated in 1962 by Frankl (1963), the theory asserts that the pursuit of existential purpose is central to psychological integrity and adaptive functioning. Conversely, the absence of perceived meaning may precipitate existential distress, often manifesting as emotional dysregulation, confusion, or psychological suffering. According to Frankl, individuals derive life meaning through three primary avenues: creative values, experiential values, and attitudinal values (Frankl, 1963). When physical or cognitive impairments disrupt an individual’s capacity to fulfill creative or experiential roles—common challenges in aging populations—the resulting loss of meaning-generating pathways may trigger affective disturbances (Frankl, 1963; Steger et al., 2006). These disturbances, particularly anxiety and depression, can propagate through interconnected biopsychosocial systems, potentially exacerbating cognitive decline and functional deterioration in older adults. In such circumstances, attitudinal values emerge as a critical compensatory mechanism, enabling individuals to reconstruct a sense of purpose through acceptance, resilience, and transcendence. This dimension reflects higher-order spiritual needs, encompassing perceptions of personal mission, existential coherence, and intrinsic worth. Notably, religious faith represents a salient component of spiritual frameworks and may serve as a structured resource for cultivating attitudinal meaning. Thus, this theoretical model provides a robust psychological rationale for examining the interplay among physical disability, affective disorders, and cognitive impairment in later life—offering insight into the underlying motivational dynamics that influence health outcomes in aging populations.

Based on the above theoretical framework and research gap, the current study addressed two aspects. First, we explored the mediating or suppressing role of anxiety and depressive symptoms on the relationship between BADLS limitations and cognitive impairment among older Chinese adults after adjusting for sociodemographic and health-related factors. Second, we compared the discrepancy of two influencing paths among older adults without religious beliefs to develop a preliminary theoretical basis for psychological interventions for cognitive function in older adults based on religion.

## Methods

### Study design and participants

**Figure 1:**
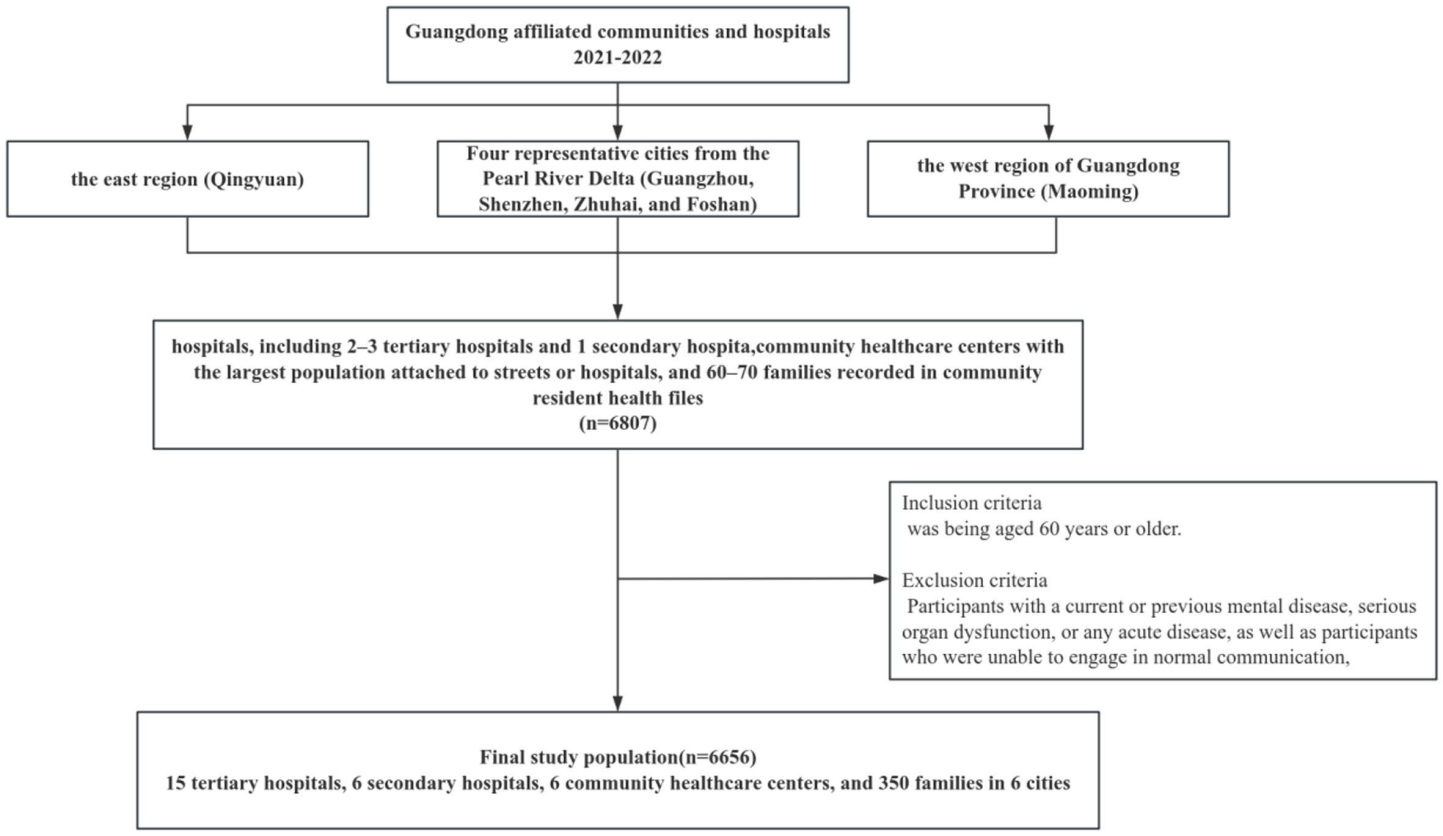
Selection process of the study population.

This population-based, cross-sectional study used a multistage, stratified, cluster-sampling procedure and was conducted between June 2021 and December 2022. This study was carried out as part of the “Active health and technological response to aging” subproject carried out by the Ministry of Science and Technology of China. The selection of study population was shown in Figure 1. Four representative cities from the Pearl River Delta (Guangzhou, Shenzhen, Zhuhai, and Foshan), one city from the east region (Qingyuan), and one city from the west region of Guangdong Province (Maoming) were selected as the main study sites. Data collection was primarily conducted at hospitals, including 2–3 tertiary hospitals and 1 secondary hospital with a reputation for providing geriatric healthcare in each city. In view of the diversity of care models for older adults in China, we also collected data from community healthcare centers with the largest population attached to streets or hospitals, and 60–70 families recorded in community resident health files to avoid selection bias. The main inclusion criteria was being aged 60 years or older. Participants with a current or previous mental disease, serious organ dysfunction, or any acute disease, as well as participants who were unable to engage in normal communication, were excluded. Ultimately, a total of 6,656 older adults from 15 tertiary hospitals, 6 secondary hospitals, 6 community healthcare centers, and 350 families in 6 cities were included in the analysis. This study was approved by the ethics committee of Guangdong Province People’s Hospital (KY-Z-2021-690-01) and all participants provided written informed consent.

### Measures

#### General participant information

General participant information was collected using a questionnaire developed based on previous studies and reviewed by two experts in a pilot study. This questionnaire included sociodemographic information (gender, age, body mass index (BMI), education level, type of residence, family income, etc.) and health-related information (chronic diseases, types of medication, drinking, smoking, regular physical examination, regular social activities, etc.).

#### Religious beliefs

We used a single question to assess religious beliefs: Do you believe in a religion (Christianity, Buddhism, Islam, etc.) or usually participate in individual or group religious-related activities? If participants answered “yes,” they were considered to have religious beliefs; otherwise, participants were considered to not have religious beliefs.

#### Cognitive impairment

Cognitive status was evaluated using the 30-item Chinese version of the Mini-Mental State Examination (MMSE). The MMSE was designed by Cockrell and Folstein (1988) in 1975 and includes dimensions of orientation, memory, attention and calculation, recall, and language. The MMSE has a sensitivity up to 92.5% to detect cognitive impairment (Bo et al., 2018). Correct responses were scored as 1, while incorrect or unclear responses were scored as 0. The MMSE is scored out of 30 points, with lower scores indicating more serious cognitive decline. The Chinese version of the MMSE demonstrated excellent internal consistency in this sample (Cronbach’s *α* = 0.914).

#### BADLs

Activities of daily living (ADLs) reflect essential activities performed by individuals living in medical institutions, communities, and families (Cornelis et al., 2019). Because IADLs items might partially overlap with early cognitive decline, we use the BADLs rating scale for ADLs assessment. BADLs included eight basic movements and self-care activities: eating, bathing, combing, dressing, controlling urine, controlling excrement, walking, and walking up and down stairs (Cornelis et al., 2019). Each item is evaluated at 2 to 4 level. The highest level is scored 15 points, and lowest level is scored 0 point. The BADLs rating scale is scored out of 100 points, with lower scores indicating worse self-care ablility, which means more serious physical limitations.

#### Anxiety and depressive symptoms

Anxiety and depressive symptoms were measured using the Generalized Anxiety Disorder-7 (GAD-7) and Health Questionnaire-9 (PHQ-9), respectively. Both scales are widely used instruments for assessing mental health. GAD-7 was used to evaluate the frequency of the following seven conditions over the previous 2 weeks: tension, uncontrollable worries, excessive worries, inability to relax, akathisia, irritability, and foreboding (Löwe et al., 2008). Each item was evaluated using a 4-point Likert scale with total scores ranging from 0 to 21. Anxiety symptoms were divided into four levels: mild (5–9), moderate (10–13), moderate–severe (14–18), and severe (19–21). PHQ-9 was used to evaluate the frequency of nine conditions over the previous 2 weeks: displeasure, appetite change, fatigue, worthlessness, guilt, decreased concentration, slow movement, restlessness, and suicidal tendency (Kroenke et al., 2001). Each item was evaluated using a 4-point Likert scale with total scores ranging from 0 to 27. Depressive symptoms were divided into four levels: mild (5–9), moderate (10–14), moderate–severe (15–19), and severe (10–27). The Cronbach’s *α* coefficient of the GAD-7 and PHQ-9 was 0.970 and 0.944, respectively, indicating excellent internal consistency in this sample.

### Data collection procedure

Data were collected using the Wechat mini program called “Jingyice platform on the functional assessment of older adults.” Written permission was obtained by each of the selected hospitals and community study sites. Formal investigations were performed by specialized interviewer teams recommended by the hospitals and communities. For door-to-door data collection from families, interviewers were screened and trained by the corresponding communities.

Unified guidelines were used when describing the study aims, obtaining informed consent, and carrying out face-to-face interviews. Consultations with caregivers or other family members chosen by subjects were allowed if older adults were unable to communicate with interviewers due to speech or hearing dysfunctions. All questionnaires were checked by the interviewers for missing data or errors. The data were stored in a secured file only accessible by authorized personnel.

### Data analysis

Data analysis was performed using SPSS software version 26.0. Quantitative variables (such as MMSE scores) are reported as the mean ± standard deviation (SD), while qualitative variables (such as gender, age stratification, and education level) are reported as absolute values and percentages. We used the *t* test or chi-square test to compare differences in MMSE scores between groups based on sociodemographic or health-related factors. Pearson analysis was utilized to examine the correlation between cognitive impairment, BADLs, anxiety, and depressive symptoms among older adults with or without religious beliefs. Hayes’ (2013) SPSS PROCESS macro software (Model 4; Igartua and Hayes, 2021) was employed to explore the independent indirect effect of anxiety and depressive symptoms on the association between BADLs limitations and cognitive impairment. To explore whether there is a serial multiple indirect effect of anxiety and depressive symptoms, we further used Hayes’ (2013) SPSS PROCESS macro (Model 6; Igartua and Hayes, 2021). We, respectively, established two path models for older adults with or without religious beliefs and then compared differences in the mediating or suppressing roles. The suppressing effect, a specific form within generalized mediation models, describes a unidirectional or bidirectional inhibitory relationship among variables. It can be interpreted as such when the direct and indirect effects hold opposing signs. Bootstrap confidence intervals (CI) at 95% and 5,000 bootstrap samples were selected for the analysis. If the boundaries between the lower and upper values of the 95%CI did not cross zero, the models were regarded as significant at the 0.05 level.

## Results

### General participant characteristics

Of the total 6,656 participants, 943 (14.17%) reported having religious beliefs and 5,713 (85.83%) reported no religious beliefs. The sample included 3,106 males (46.66%) and 3,550 females (53.34%). The average age was 71.97 ± 8.10 years; 46.06% of participants were in the range of 60–69 years old. The majority of participants (56.46%) had a standard BMI. More than half of participants had an education level of primary school or below (67.64%), more than three children (65.69%), were married (85.08%), and lived with family members (96.62%). Regarding region of residence, 53.80 and 46.20% of participants resided in urban and rural regions, respectively. Most participants received rural medical services (67.71%) and had a family income ranging from 2000–4,000¥ per month (44.62%). Regarding health-related factors, nearly 20 and 10% of participants had more than three chronic diseases and took four types of medication, respectively. A small proportion of participants drank alcohol (6.51%), while the majority of participants smoked (87.58%). More than half of participants regularly participated in social activities (54.46%) and exercised (59.03%), while a small proportion (41.56%) regularly received physical examination by healthcare providers. As shown in Table 1, univariate analysis indicated a significant discrepancy in cognitive function between subgroups of each general characteristic (*p* < 0.001) for older adults with religious beliefs except for dwelling state (*p* = 0.056). There was also a significant discrepancy in cognitive function between subgroups without religious beliefs (*p* < 0.001).

**Table 1:** Basic characteristics related to cognitive function among older adults with or without religious belief (*n* = 6,656).

Variables	N (%)	With religious belief (*n* = 943)			Without religious belief (n = 5,713)		
		MMSE (Mean±SD)	*t*/*F*	*p* value	MMSE (Mean±SD)	*t*/*F*	*p* value
Gender			−5.177	<0.001**		4.615	<0.001**
Male	3,106 (46.66)	14.35 ± 8.34			23.06 ± 6.40		
Female	3,550 (53.34)	17.20 ± 8.44			22.25 ± 6.77		
Age, years old			84.239	<0.001**		466.624	<0.001**
60–69	3,066 (46.06)	21.14 ± 8.68			24.64 ± 4.99		
70–79	2,305 (34.63)	14.94 ± 7.79			22.22 ± 6.42		
≥80	1,285 (19.31)	12.64 ± 7.09			17.80 ± 8.15		
BMI^a^, kg/m^2^			42.840	<0.001**		92.139	<0.001**
<18.5	582 (8.74)	20.19 ± 9.01			19.69 ± 7.86		
18.5–23.9	3,758 (56.46)	14.37 ± 7.95			22.01 ± 7.01		
24–27.9	1900 (28.55)	21.13 ± 8.23			24.23 ± 5.18		
>28	416 (6.25)	24.52 ± 5.59			24.36 ± 4.95		
Education level			87.801	<0.001**		172.728	<0.001**
Primary school and below	4,502 (67.64)	14.33 ± 7.92			21.33 ± 6.99		
Junior high school	1,156 (17.37)	25.86 ± 4.22			24.88 ± 5.06		
Senior high school	792 (11.90)	25.66 ± 4.89			25.66 ± 4.99		
College and above	206 (3.09)	26.40 ± 2.41			25.13 ± 5.54		
Marital status			19.140	<0.001**		22.566	<0.001**
Married	5,663 (85.08)	15.35 ± 8.34			22.87 ± 6.48		
Widowed	923 (13.87)	20.67 ± 8.40			21.21 ± 7.18		
Other conditions^b^	70 (1.05)	22.11 ± 8.78			23.13 ± 7.12		
Quantities of children			53.760	<0.001**		58.658	<0.001**
None	141 (2.12)	19.72 ± 9.01			20.05 ± 7.48		
1	564 (8.47)	24.19 ± 5.24			24.56 ± 5.87		
2	1,579 (23.72)	24.10 ± 5.70			23.96 ± 5.55		
≥3	4,372 (65.69)	14.60 ± 8.15			21.88 ± 6.93		
Types of residence			−18.455	<0.001**		−19.392	<0.001**
Urban regions	3,581 (53.80)	24.81 ± 4.92			20.64 ± 7.42		
Rural regions	3,075 (46.20)	13.77 ± 7.76			23.99 ± 5.62		
Living alone or not			1.914	0.056		−2.670	0.008**
Yes	225 (3.38)	19.21 ± 7.89			21.41 ± 7.26		
No	6,431 (96.62)	15.85 ± 8.51			22.68 ± 6.59		
Types of insurance			68.747	<0.001**		62.889	<0.001**
Rural medical service	4,507 (67.71)	14.85 ± 8.26			21.91 ± 6.95		
Urban medical service	1749 (26.28)	25.25 ± 4.51			23.91 ± 5.84		
Other medical service	400 (6.01)	17.56 ± 7.49			24.09 ± 5.16		
Family income per month, ¥			163.306	<0.001**		106.605	<0.001**
<2000	1,086 (16.32)	21.33 ± 7.35			21.49 ± 6.34		
2000–4,000	2,970 (44.62)	13.48 ± 7.71			21.57 ± 7.28		
>4,000	2,600 (39.06)	24.27 ± 5.36			24.08 ± 5.74		
Quantities of chronic diseases			15.543	<0.001**		9.088	<0.001**
<3	5,490 (82.48)	19.66 ± 8.31			22.93 ± 6.32		
≥3	1,166 (17.52)	11.97 ± 6.75			20.53 ± 8.08		
Types of medication			28.493	<0.001**		3.673	0.005**
None	2,738 (41.14)	16.79 ± 9.58			22.54 ± 6.76		
1	1,468 (22.06)	13.10 ± 7.05			22.69 ± 6.34		
2	1,189 (17.86)	19.53 ± 7.73			23.13 ± 6.14		
3	615 (9.24)	19.56 ± 7.91			22.79 ± 6.61		
≥4	646 (9.70)	21.36 ± 6.74			21.88 ± 7.21		
Drinking			−4.936	<0.001**		−7.389	<0.001**
Yes	433 (6.51)	24.29 ± 5.51			24.95 ± 4.99		
No	6,223 (93.49)	15.71 ± 8.47			22.45 ± 6.69		
Smoking			−5.869	<0.001**		−9.299	<0.001**
Yes	5,829 (87.58)	23.33 ± 5.81			24.65 ± 4.84		
No	827 (12.42)	15.59 ± 8.46			22.31 ± 6.80		
Regular physical examination			−12.493	<0.001**		−15.425	<0.001**
Yes	2,766 (41.56)	21.70 ± 5.96			24.11 ± 5.11		
No	3,890 (58.44)	14.15 ± 8.39			21.45 ± 7.40		
Regular social activities			−19.818	<0.001**		−25.850	<0.001**
Yes	3,625 (54.46)	22.42 ± 6.14			24.46 ± 4.64		
No	3,031 (45.54)	12.65 ± 7.61			20.12 ± 7.67		
Regular exercise			−20.739	<0.001**		−28.283	<0.001**
Yes	3,929 (59.03)	22.19 ± 6.44			24.42 ± 4.75		
No	2,727 (40.97)	12.30 ± 7.39			19.62 ± 8.06		

a Body mass index.

b Other conditions mean spinsterhood or divorce.

**p < 0.01. The bold values is used to highlight key data in the tables.

### Correlation analysis

Table 2 presents the correlations between BADLs, anxiety symptoms, depressive symptoms, and cognitive function. For older adults with and without religious beliefs, the correlations between all variable pairs were significant (all *p* < 0.001). BADLs were positively associated with cognitive function, while anxiety symptoms were positively related to depressive symptoms. Both BADLs and cognitive function were negatively associated with anxiety and depressive symptoms.

**Table 2:** The correlation analysis between cognitive impairment, basic activities of daily living, anxiety symptoms and depressive symptoms among older adults with or without religious belief (*n* = 6,656).

Groups	Variables	Mean ± SD	Cognitive impairment	BADLs	Anxiety symptoms	Depressive symptoms
With religious belief	Cognitive impairment	15.93 ± 8.51	—	0.532**	−0.538**	−0.529**
	BADLs	80.92 ± 28.94		—	−0.098**	−0.095**
	Anxiety symptoms	7.64 ± 5.19			—	0.910**
	Depressive symptoms	9.55 ± 7.05				—
Without religious belief	Cognitive impairment	22.63 ± 6.61	—	0.665**	−0.274**	−0.343**
	BADLs	88.92 ± 25.83		—	−0.271**	−0.336**
	Anxiety symptoms	3.08 ± 4.10			—	0.850**
	Depressive symptoms	3.51 ± 4.92				—

BADLs, Basic activities of daily living.**p < 0.001.

### The suppressing effect of anxiety symptoms and mediating effect of depressive symptoms

The PROCESS model 4 was used to explore the mediating or suppressing effect of depressive and anxiety symptoms on the correlation between BADLs and cognitive impairment. Gender, age, BMI, education level, marital status, number of children, residence type, living alone or not, insurance type, family income, quantities of chronic diseases, types of medication, drinking, smoking, regular physical examination, regular social activities, and regular exercise were included as covariates. For older adults with religious beliefs, the total, direct, and indirect effect models were significant (*p* < 0.001). BADLs were significantly associated with anxiety symptoms (*β* = 0.019, SE = 0.005, *p* < 0.001) and cognitive impairment (*β* = 0.112, SE = 0.007, *p* < 0.001). Anxiety symptoms were also significantly associated with cognitive impairment (*β* = −0.341, SE = 0.043, *p* < 0.001). Similarly, BADLs were significantly associated with depressive symptoms (*β* = 0.029, SE = 0.007, *p* < 0.001) and cognitive impairment (*β* = 0.112, SE = 0.007, *p* < 0.001). Depressive symptoms were also significantly associated with cognitive impairment (*β* = −0.225, SE = 0.033, *p* < 0.001). There was a suppressing role of anxiety (*β* = −0.007, SE = 0.002, 95%*CI*: −0.0011, −0.003) and depressive symptoms (*β* = −0.007, SE = 0.002, 95%*CI*: −0.011, −0.003) on the correlation between BADLs and cognitive impairment, each of which accounted for approximately 6.67% of variance in the total effect.

The same approach was used to explore relationships between variables among participants without religious beliefs. The results showed that the total, direct, and indirect effect models were significant (*p* < 0.001). BADLs were significantly associated with anxiety symptoms (*β* = −0.028, SE = 0.002, *p* < 0.001) and cognitive impairment (*β* = 0.138, SE = 0.003, *p* < 0.001). Anxiety symptoms were also significantly associated with cognitive impairment (*β* = −0.095, SE = 0.016, *p* < 0.001). Similarly, BADLs were significantly associated with depressive symptoms (*β* = −0.044, SE = 0.003, *p* < 0.001) and cognitive impairment (*β* = 0.135, SE = 0.003, *p* < 0.001). Depressive symptoms was also significantly associated with cognitive impairment (*β* = −0.118, SE = 0.014, *p* < 0.001). Anxiety (*β* = 0.003, SE = 0.006, 95%*CI*: 0.002, 0.004) and depressive symptoms (*β* = 0.005, SE = 0.001, 95%*CI*: 0.004, 0.007) partially mediated the effect of BADLs on cognitive impairment, each of which accounted for approximately 2.14 and 3.57%, respectively, of variance in the total effect.

### Chained mediating or suppressing effect of anxiety and depressive symptoms

**Figure 2:**
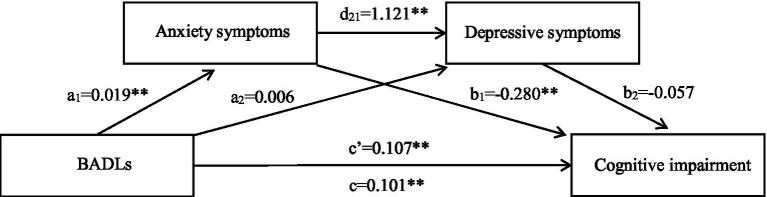
Chained mediation effects model of mediating or masking effect of anxiety and depressive symptoms on association between BADLs and cognitive impairment among individuals who have religious belief. c, Total effect; c’, Direct effect; a_1,_ Effect of BADLs on anxiety symptoms; a_2_, Effect of BADLs on depressive symptoms; b_1_, Effect of anxiety symptoms on cognitive impairment; b_2_, Effect of depressive symptoms on cognitive impairment; d_21_, Effect of anxiety on depressive symptoms; BADLs, Basic activities of daily living. **p < 0.01.

A chained mediation effects model was established using PROCESS model 6. The regression analysis results are shown in Table 3. For participants with religious beliefs, the direct effect of BADLs on anxiety symptoms (*β* = 0.019, *p* < 0.001) and cognitive impairment (*β* = 0.107, *p* < 0.001), and the effect of anxiety on depressive symptoms (*β* = 1.121, *p* < 0.001) and cognitive impairment (*β* = −0.280, *p* < 0.001), were significant. However, there was no significant impact of BADLs on depressive symptoms (*β* = 0.006, *p* = 0.084), or of depressive symptoms on cognitive impairment (*β* = −0.057, *p* < 0.334). As shown in Table 4 and Figure 2, there was a significant total effect (*β* = 0.101, SE = 0.007, 95%*CI*: 0.088, 0.0114) and direct effect (*β* = 0.107, SE = 0.007, 95%*CI*: 0.095, 0.121) of BADLs on cognitive impairment. Anxiety symptoms played an independent suppressing role in the relationship between BADLs and cognitive impairment (*β* = −0.006, SE = 0.002, 95%*CI*: −0.011, −0.003), whereas depressive symptoms did not mediate the path from BADLs to cognitive impairment.

**Table 3:** The results of regression analysis in chained mediation effects model (*n* = 6,656)^a^.

Groups	Dependent variables	Independent variables	*R*	*R* ^2^	*F*	Standardized *β*	*β*	*t*	*p*
With religious belief	Anxiety	BADLs	0.673	0.453	42.450	0.103	0.019	3.645	<0.001**
	Depressive symptoms	BADLs	0.919	0.844	262.568	0.026	0.006	1.729	0.084
		Anxiety symptoms	—	—	—	0.825	1.121	46.952	<0.001**
	Cognitive impairment	BADLs	0.811	0.657	88.305	0.366	0.107	16.174	<0.001**
		Anxiety symptoms	—	—	—	−0.171	−0.280	−3.563	<0.001**
		Depressive symptoms	—	—	—	−0.047	−0.057	−0.966	0.334
Without religious belief	Anxiety	BADLs	0.355	0.126	45.755	−0.171	−0.027	−11.988	<0.001**
	Depressive symptoms	BADLs	0.860	0.740	852.793	−0.088	−0.017	−11.122	<0.001**
		Anxiety symptoms				0.808	0.970	111.753	<0.001**
	Cognitive impairment	BADLs	0.726	0.527	316.816	0.525	0.135	48.809	<0.001**
		Anxiety symptoms				0.040	0.064	2.271	0.023*
		Depressive symptoms				−0.121	−0.162	−6.756	<0.001**

a Final regression model adjusted for all covariates.

BADLs: Basic activities of daily living.*p < 0.05, **p < 0.01. The bold values is used to highlight key data in the tables.

**Table 4:** The relationship between basic activities of daily living and cognitive impairment with mediating or masking role of anxiety and depressive symptoms among older adults with our without religious belief (*n* = 6,656).

Groups	Pathways	Effect	Boot SE	95%*CI*	Proportion of effect(%)
With religious belief	Total effect: BADLs→cognitive impairment	0.101*	0.007	(0.088, 0.114)	100.00
	Direct effect: BADLs→cognitive impairment	0.107*	0.007	(0.095, 0.121)	105.94
	Indirect effect: BADLs→cognitive impairment	−0.006*	0.002	(−0.011, −0.003)	5.94^a^
	Indirect effect 1: t BADLs→anxiety symptoms→cognitive impairment	−0.005*	0.002	(−0.010, −0.002)	4.95
	Indirect effect 2: BADLs→depressive symptoms→cognitive impairment	−0.0004	0.001	(−0.002, 0.0004)	0.16
	Indirect effect 3: BADLs→anxiety symptoms→depressive symptoms→cognitive impairment	−0.001	0.001	(−0.004, 0.002)	0.83
Without religious belief	Total effect: BADLs→cognitive impairment	0.140*	0.003	(0.134, 0.145)	100.00
	Direct effect: BADLs→cognitive impairment	0.135*	0.003	(0.129, 0.140)	96.43
	Indirect effect: BADLs→cognitive impairment	0.005*	0.001	(0.004, 0.007)	3.57
	Indirect effect 1: BADLs→anxiety symptoms→cognitive impairment	−0.002*	0.001	(−0.004, −0.0001)	1.43^b^
	Indirect effect 2: BADLs→depressive symptoms→cognitive impairment	0.003*	0.001	(0.002, 0.004)	2.14
	Indirect effect 3: BADLs→anxiety symptoms→depressive symptoms→cognitive impairment	0.004*	0.001	(0.003, 0.006)	2.86

^a^Indirect effect is regarded as masking effect calculated by |c’/c|.

^b^Indirect effect 1 is regarded as masking effect calculated by |a_1_b_1_/c-c’|.

BADLs, Basic activities of daily living. *Empirical 95% confidence interval dose not include zero. The bold values is used to highlight key data in the tables.

**Figure 3:**
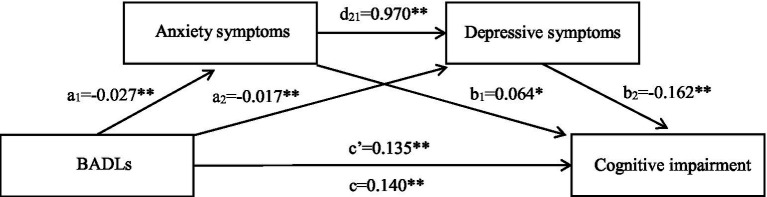
Chained mediation effects model of mediating or masking effect of anxiety and depressive symptoms on association between BADLs and cognitive impairment among individuals who do not have religious belief. c, Total effect; c’, Direct effect; a_1_, Effect of BADLs on anxiety symptoms; a_2_, Effect of BADLs on depressive symptoms; b_1_, Effect of anxiety symptoms on cognitive impairment; b_2_, Effect of depressive symptoms on cognitive impairment; d_21_, Effect of anxiety on depressive symptoms; BADLs, Basic activities of daily living. *p < 0.05; **p < 0.01.

For participants without religious beliefs, the total and direct effects of BADLs on cognitive impairment remained significant (*p* < 0.05; Tables 3, 4). All of the indirect impacts, which differed from those for participants with religious beliefs, were statistically significant in the path from BADLs to cognitive impairment (*p* < 0.001; Table 4; Figure 3). Of the indirect effect, anxiety symptoms still played a suppressing effect in the association between BADLs and cognitive impairment (*β* = −0.002, SE = 0.001, 95%*CI*: −0.004, −0.0001). Depressive symptoms partially mediated the effect of BADLs on cognitive impairment (*β* = 0.003, SE = 0.001, 95%*CI*: 0.002, 0.004). The impact path from BADLs to cognitive impairment was sequentially mediated by anxiety and depressive symptoms (*β* = 0.004, SE = 0.001, 95%*CI*: 0.003, 0.006).

## Discussion

In this population-based, cross-sectional study of older Chinese adults, we found that, compared to participants without BADL limitations, those with BADL limitations reported significantly worse cognitive function regardless of whether they had religious beliefs. Furthermore, the multiple mediating effect model results revealed that the association between BADLs limitations and cognitive impairment may in part be attributed to depressive symptoms among older adults without religious beliefs, whereas a mediating effect of depression was not found among older adults with religious beliefs. Notably, anxiety and depressive symptoms sequentially mediated the association between BADL limitations and cognitive impairment. We also found that anxiety symptoms played an independent suppressing role on the effect of BADLs on cognitive decline among older adults with or without religious beliefs.

There is a broad consensus that physical dysfunction is significantly related to cognitive decline (Zhang and Yang, 2024; de Paula et al., 2015; Biazus-Sehn et al., 2020; Zhang et al., 2024), although the mechanisms remain controversial (Peng et al., 2023). Previous studies (Kojima et al., 2016; Angevaren et al., 2008; Basso and Suzuki, 2017; Ratey and Loehr, 2011) have speculated that older adults with lower ADLs have worse physical activities and bodily function, which may be explained by impaired cardiopulmonary fitness, weakened neurotransmitters, and increased stress, ultimately leading to decreased cognitive function. Frailty or pre-frailty, which occurs with the aging process, has also been shown to be a predictive factor for physical impairment in prior studies (Pegorari and Tavares, 2014; Wu et al., 2017), which is considered one aspect of “cognitive frailty” (Kojima et al., 2016; Kelaiditi et al., 2013). Consistent with prior reports (Peng et al., 2023; Hammar et al., 2022; Dotson et al., 2020; Ahern et al., 2024), we found that a negative mental state like anxiety or depression is linked to cognitive impairment. This correlation is thought to share the same underlying pathophysiology mechanisms (Hammar et al., 2022; Rock et al., 2014), such as changes in white matter microstructure (Chen et al., 2021), although more biomedical evidence is needed (Peng et al., 2023). In conclusion, ADLs limitations and psychological disorders may be important predictive indexes of cognitive impairment with a similar influencing path, as proposed by Li and Xu (2024).

Furthermore, our study explored the possible mechanism of the effect of BADL limitations on cognitive impairment from a psychological perspective. Although the current findings suggest that depressive disorders may aggravate cognitive function declines among older adults with basic physical activity restriction (Wu, 2021; Peng et al., 2023; Zhang and Yang, 2024), the proportion of explained variance in the total association was less than 5%, which was significantly lower than the proportions reported by Li and Wu (2022) and de Paula et al. (2015). A possible explanation of this difference is that the above two studies used multi-dimensional assessment methods to measure cognitive function, such as execution, memory, language, and visuospatial abilities, rather than single instrument, resulting in examination of more complex aspects of the association between mental health and cognitive impairment. Moreover, a 6-year longitudinal study by Li and Wu (2022) fully confirmed the causal relationship between variables. This might also because the association between physical and cognitive function is mediated by diverse factors such as activity knowledge, skills, social network, social support, and multiple-disease states according to Human Capital Model (Bailey et al., 2012). However, only a part of these potentially influential factors were adjusted for in our study when established mediation effects model. Accordingly, we should adopt longitudinal design or measurement methods, including those of specific cognitive features, and explore the mediating effect from other individual or social factors, to broadly explain the effect of ADL limitations on cognitive impairment beyond the mental aspect.

Despite the finding of a weak indirect effect of depressive symptoms in this study, the partial mediating role in pathway was significant. This conclusion supports the fundamental mechanism proposed by the theory of life meaning—when primary sources of meaning are disrupted, individuals may fall into emptiness and helplessness, manifesting as despair and fear regarding lost capabilities and an uncertain future, which in turn triggers psychological distress. The mechanism underlying this association may be linked to the fact that older adults with physical limitations lack the energy to participate in external interactions with friends and nearby people (Li and Xu, 2024; Liao and Chang, 2020; Wassink-Vossen et al., 2014), leading to lower social communication and support. Previous studies (Lee et al., 2022; Wen et al., 2023) have found that older adults with lower social interactions report more negative mental health and may develop depression. Older people with psychological deficits are speculated to suffer brain structure and nerve function impairments, which can be explained by various hypotheses such as impaired neuroplasticity of the medial prefrontal cortex or hippocampus (Naismith et al., 2012), memory deficits, and the vascular hypothesis (Butters et al., 2008), all of which increase the occurrence of cognitive impairment. Moreover, low social participation is predictive of emotional disturbance among older adults, which is related to functional status deterioration (Wong et al., 2024).

Our study also investigated the mediating effect of depressive symptoms on the correlation between physical and cognitive impairment among older adults with or without religious beliefs. The results suggest that individuals without religious beliefs who suffer basic self-care disability may experience a higher risk of cognitive decline through the negative influence of depressive status. In contrast, no significant effect of depressive symptoms was seen in the religious group. These findings suggest a crucial protective value of religious and spiritual beliefs on mental health, consistent with the findings of a meta-analysis of 102 observational studies (Coelho-Júnior et al., 2022) and a systematic review of 152 prospective studies (Braam and Koenig, 2019). There are several possible explanations for this finding. Firstly, religious activities might contribute to more positive social interactions and strengthen mental health, as discussed in similar studies (Nath et al., 2023; Lee and Kim, 2016; Park et al., 2024). Older adults who participate in religious activities more frequently show more self-confidence in coping with adverse life events by acquiring guidance from religious organizations or peer support (Coelho-Júnior et al., 2022; Dein, 2006). Secondly, from an internal perspective, religion might relieve depressive symptoms by providing meaning and increasing psychological resilience when someone faces a dilemma, promoting a transcendent approach that changes one’s attention to other people’s happiness instead of excessively focusing on the self (Lee and Newberg, 2005). In addition, Maranges and Fincham (2024) found that religious beliefs might provide a connection between self-control and lower mental distress.

Religion is traditionally regarded as a multidimensional construct dependent on the synthesis of beliefs (Coelho-Júnior et al., 2022), public involvement (Rajkumar, 2021), private practices (Lee and Kim, 2016), and intrinsic mental or cognitive aspects like religious coping and motivation (Maranges and Fincham, 2024). However, the assessment of religious beliefs in our study was designed to single question and binary answers, which cannot fully consider the complex forms of religion. Numerous studies (Zhang et al., 2024; Kobayashi et al., 2020) examining the interrelation of religion and depression have reported considerable variation between continents or countries, highlighting the necessity to consider the effect of regional heterogeneity on religious connotation. For example, Chinese Buddhist culture has been proven to be valuable for relieving the adverse impact of perceived inequality and pressure from traditional social attributes on subjective well-being (Tan et al., 2023). However, partial studies (Aggarwal et al., 2023; Szcześniak and Timoszyk-Tomczak, 2020) conducted in western countries with a profound Christian culture or background have described a concept of negative religious coping, called “religious struggle,” which is associated with depression development. Therefore, future studies should focus on a common mechanism of psychological and cognitive disorder based on particular aspect of religion. Furthermore, the multifaceted nature of Chinese cultural traditions and sociocultural constructs—such as filial piety, collective coping strategies, and secular moral beliefs—should be fully considered in relation to religious faith, thereby enriching the conceptualization of religious involvement within the sociocultural context of China.

Another major finding of our study is that anxiety symptoms play a significant suppressing effect on the association between BADL limitations and cognitive impairment among older adults. A partial effect and a total suppressing effect in indirect effect, respectively, were found in the religious and non-religious groups. These different findings suggest that the indirect effect via anxiety symptoms might weaken the total direct effect of physical limitations on cognitive impairment. Compared to depressive symptoms, anxiety might alleviate the adverse impact of limited physical activities on cognitive impairment. These findings are inconsistent with prior reports in the psychosomatic field that mental disorders harm physical or cognitive health (Smith et al., 2021; Hu et al., 2020). These differences could be due to the fact that anxiety, especially an early anxiety state, is presumed to be an adaptive strategy contributing to positive stress coping styles and adaptive behaviors (Micoulaud-Franchi et al., 2024; Giovanniello et al., 2023). Individuals often show adaptive effects during the early period of mild stress after a negative event, which is commonly described as hypervigilance and includes different manifestations like enhanced selective attention (Hoskin et al., 2014), shorter response delay (Shields et al., 2019), and stronger working memory (Henckens et al., 2009; Yuen et al., 2009). This process may improve associative learning and adaptive feedback in the short term, and help advance disease management (Shields et al., 2016). However, employing the suppressing effect to interpret the role of anxiety between physical and cognitive dysfunction carries certain risks. Firstly, although the suppressing effect was statistically significant, the total effect remained significant despite the counteraction of the direct effect. This indicates that the statistical explanatory power of anxiety’s suppressing effect is limited. Secondly, existing evidence does not show an overall beneficial influence of anxiety on pressure coping due to the complexity of mental health conditions intensity, measurements, and the environment in which stress experience (Giovanniello et al., 2023). Further research is needed to understand the evolutionary mechanism of psychologies inducing disease formulation in the future.

### Implications and limitations

There is a lack of religious studies in China because of the low popularity of religious concepts in Chinese societies (Zhang et al., 2024). Individuals with religious beliefs may be considered to have social values deviating from the norm, and may be stigmatized or marginalized (Zhang et al., 2024). Hence, health professionals lack basic knowledge and experience in this field (Peteet et al., 2019), even though it may be essential for systematic disability management among older people. In light of this, the present study offers perspectives beyond existing research that religious belief has a direct impact on the association between mental health and disability status among older Chinese adults.

These findings are encouraging and serve as a reminder that religious beliefs may play a more important role than originally thought, providing health professionals with new directions related to psychological management of disabled older adults. More attention should be paid to the role of depressive disorder in mediating the correlation between physical limitations and cognitive impairment among older adults without religious beliefs. Moreover, while ensuring regulatory compliance, due attention should be given to the positive role of religious belief in promoting mental health and mitigating functional decline. There is a need to systematically identify and integrate doctrinal elements that align with evidence-based health promotion principles, thereby guiding religion to become a constructive component of aging management strategies. When designing psychosocial and cognitive interventions for elderly populations, healthcare providers should acknowledge the potential contribution of religious dimensions and account for the heterogeneity across religious affiliations and practices in shaping health outcomes. The development and implementation of culturally adapted, context-sensitive religiously framed interventions—grounded in the sociocultural fabric of Chinese society—are therefore both justified and necessary.

There are some limitations that restrict our findings. First, the cross-sectional design limits our ability to draw causal explanations or understand the temporal precedence in which variables interact. Prospective studies should be conducted to explore the causal relationship between related factors. Second, although we established a chained mediating effect model adjusted for probable confounding factors and found an indirect effect of anxiety and depressive symptoms on the association between ADL limitations and cognitive impairment, this accounted for a small proportion of the total effect. Thus, other variables may play a regulating role in the paths. Further research should include more variables to explore multiple mechanisms influencing disability according to a mature theoretical framework. Third, the current study only used a single question to identify participants with or without religious beliefs. The ratio of individuals with religious affiliation to those without was 1:6, indicating an imbalanced sample distribution that may have attenuated statistical power to some extent. Due to the complexity of religious categories and connotations, evaluation systems consisting of additional religion forms in accord with regional culture should be developed in the future. Fourth, Although this study adjusted for several sociodemographic and behavioral covariates, potential confounders that may jointly influence both religious involvement and cognitive function—such as social participation and household composition—were not included in the analyses. This limitation may reduce the interpretability of the observed associations. Future research should incorporate a broader set of theoretically relevant variables and employ advanced modeling techniques to elucidate the complex interplay among these factors. Last but not least, only MMSE was used to assess cognitive impairment. A more comprehensive approach including systematic function aspects, such as language, memory, or execution function, and professional neurological examination is warranted to confirm diagnosis.

## Conclusion

The findings of the current study suggest that physical limitations in older Chinese adults might influence cognitive function through the mediating role of depressive disorder and the suppressing role of anxiety. These paths were found to differ significantly between older adults with and without religious belief. Based on our findings, future therapeutic strategies aimed at addressing physical function and relieving mental health issues may aid in resolving cognitive concerns. Such treatments should focus on the prevention of depressive disorders, the potential positive effect of anxiety, and postponement of mental disorders and cognitive deterioration through religious thinking.

## Data Availability

The datasets presented in this study can be found in online repositories. The names of the repository/repositories and accession number(s) can be found in the article/supplementary material.
